# Supplementary material for: Plants Used in Mexican Traditional Medicine for the Management of Urolithiasis: A Review of Preclinical Evidence, Bioactive Compounds, and Molecular Mechanisms
Source: Molecules. 2022 Mar 21;27(6):2008. doi: 10.3390/molecules27062008 (PMC8949565; doi:10.3390/molecules27062008)
Supplement: Supplementary file 1 [file molecules-27-02008-s001.zip › molecules-1626767 Supplementary.pdf]

Supplementary Table S1. Traditional consumption and dose of medicinal plants for urolithiasis.

Common name	Binomial nomenclature	Traditional consumption and dose	Reference
Chicalote amarillo	*Argemone mexicana L*	This plant is consumed as infusion. Traditional dose: Three grams in a liter of water, it is drunk three cups a day for periods not exceeding 10 days.	[78], ([79], pp. 48-49)
Palo amarillo	*Berberis trifoliata Hartw. Ex Lindl*	There are not documented evidence about consumption or doses of this plant. Traditional dose: Not available	Not available
Planta de insulina	*Costus mexicanus Liebm*	This plant is consumed as tea or infusion. Traditional dose: Not available	[80]
Cenizo	*Chenopodium album L.*	This plant is consumed as infusion Traditional dose: Not available	[45]
Visnaga	*Ammi visnaga (L.) Lam.*	Consumed through preparations made from the decoction and/or powdered plant. Traditional dose: Not available	[47]
Palo azul	*Eysenhardtia polystachya (Ortega) Sarg*	This plant is consumed as tea. Traditional dose: One or two pieces of stem in one liter of water.	[81, 82]
Doradilla	*Selaginella lepidophylla (Hook. & Grev.) Spring*	This plant is consumed as tea or infusion. Traditional dose: Three leaves of the plant in a liter of water, it is drunk daily after meal.	[83, 84]
Dandelion	*Taraxacum officinale L*	This plant is consumed as tea or infusion. Traditional dose: Six grams of whole plant in a liter of water. Drink one cup each six hours for 25 days.	([79], pp. 58-59), [86]
